# Optimal allocation of multi-type FACTS devices for mitigating wind power spillage with enhancing voltage stability and social welfare

**DOI:** 10.1038/s41598-023-44977-9

**Published:** 2023-10-19

**Authors:** Samaa Fawzy, Elhossaini E. Abd-Raboh, Abdelfattah A. Eladl

**Affiliations:** Electrical Engineering Department, Faculty of Engineering, Mansoura University, El-Mansoura, 35516 Egypt

**Keywords:** Electrical and electronic engineering, Energy infrastructure

## Abstract

Most of countries around the world tends to increases the penetration of renewable energies generation in electrical power networks. This led to the emergence of many challenges in these systems, such as congestion of lines, voltage instability, etc. The most important of these problems is the spillage of renewable energies in order to maintain the stability of the power system. However, by using the traditional methods to mitigate the spillage, the stability of the power system may be deteriorated leading to a vulnerable power system against disturbances. This paper proposes a bilevel multi-objective Musical Chairs optimization algorithm for optimal allocation of multi-type flexible AC transmission system (FACTS) devices. The main target of the upper-level is to reduce the wind power spillage with minimize the investment cost of FACTS devices and load shedding, while maximize the voltage stability. Moreover, under different operating scenarios, the lower-level problem captured the market clearing with maintain the system constraints for maximize the social welfare. This leads to a robust and economical operating point where included enough levels of voltage security. The technique proposed in this paper is tested on the IEEE 24-bus modified reliability test system. The results show that the applicability of the proposed algorithm in aiding power system improvement planning for minimizing wind power spillage to integrate wind energy with maximizing the social welfare and improving the loadability and the voltage stability.

## Introduction

One of the most efficient approaches used by the power industry to minimize greenhouse gas emissions and achieve sustainability is the wind energy. Countries such as China and Germany require the system operators to select renewable energy, especially wind power (WP) to reduce the environmental impact and to resist the energy crisis. Many nations have established their own goals for future levels of WP penetration. For instance, by 2030 and 2050 in U.S., more than 20% and 30% of the electricity demand will be provided by WP, respectively^1^.

Due to the integration of WP into the existing electrical power grids, there are two technical challenges. The first one is related to the regions with a large availability of wind resources which may be far from the conventional generation plants or load centers. While the second one is associated with a large capacity and the variation of WP output, because the transmission system may not be designed to handle the huge power generated. Due to the insufficient capacity of transmission lines (TLs) to deliver large amounts of WP to the load centers from the remote areas, wind power spillage (WPS) will be occurred. WPS refers to the amount of the WP production which is not used due to insufficient transmission capacity^2^ or system constraints. So, WPS can be calculated as the difference between the scheduled and the available WP.

Furthermore, the power demand has increased due to the technological development in the electricity market, which has also brought many social benefits. Meanwhile, the existing TLs could not cope with increasing power demand and high penetration of renewable energies. As a result, the electrical networks are getting closer to their limits resulting in endangering the system’s security due to the congestions and critical situations. Systems can be exposed to the instabilities due to conventional planning, operating environment, and operating methods. But, one of these instabilities is the voltage instability which has resulted in a major blackout. So, during the planning and operation of deregulated power systems, voltage collapse and stability have become one of the major concerns^3^. Due to this, one of the issues that the system operator has in the electricity market is the study of improving the existing power network transfer capability to ensure its secure operation, improve social welfare (SW), and satisfy the rising power demand and renewable energies^4^.

The construction of new TLs is an approach to facilitate these problems by enhancing its capacity. However, due to the strict environmental permissions, high investment cost, and long construction time, these projects are unattractive^5^. The installation of power electronic-based controllers such as flexible AC transmission systems (FACTS) on selected lines is an economical approach to regulate the power flow in the existing TLs and improve their utilization^6^. Due to the installation of the FACTS devices (FDs) the line flow on fully loaded transmission branches can be reduced, which would lead to improving the security, enhanced loadability of the power system, reducing the WPS, and maximizing the SW. By shifting the power to the underutilized lines rather than the congested lines, transmission bottlenecks can be avoided and this occurs due to the control that occurred in the power flow by FDs. In addition, to accommodate the stochastic nature of WP, FDs can be instant dynamically adjusted, due to their fast operations^2^.

In recent years, enormous attention has been devoted to the concept of utilizing flexibility in the power system by using FDs. For example, the authors in^7^ investigated the utilization of multi-type FDs, such as static VAR compensator (SVC) and thyristor-controlled series capacitor (TCSC), during a highly stressed network to maximize power transfer transactions, and this simulates a load increase under TL and/or thermal limit outage. Also, in the near future, it is predicted that more FDs will be commercially available at significantly lower prices under the Green Electricity Network Integration (GENI) program efforts^8,9^. Therefore, to reduce the WPS and improve the integration of the WP, effective planning models that offer useful information about the optimal allocations for FDs should be established.

To minimize the WPS and maximize the utilization of wind energy, there are several articles that use single or multiple types of FDs. TCSC can be used successfully to reduce operating costs and wind power curtailment (WPC)^10^. Using TCSC, different optimal power flow (OPF) based models are proposed to overcome the security problems and system congestion^11,12^. The upper-level problem in^13^ aimed to reduce the investment cost of series FDs such as TCSC, possible load shedding (LS), and the cost of WPC. While under different operating scenarios, the lower-level problem captured the market clearing. The numerical results in^13^ illustrated the significant benefits of TCSC in the WP integration. The main objectives of^14,15^ are the minimization of the SVC operation cost, WPC, and the total social cost. To increase the integration of WP in a weak distribution network, an evaluation search about the capability of SVC to compensate for the power is presented^16^. However, these articles didn’t consider the effect of voltage stability in the objective function.

The penetration WP level is increased by effectively monitoring the active power flows using an optimal phase shifter transformer (PST)^17^. The security-constrained unit commitment (UC) is used to reduce the curtailment significantly. Moreover, a unified power flow controller (UPFC) incorporates the functionality of both series and shunt controllers to create a more economic UC schedule, and largely reduce both WPC and LS^18^. However, the SW is not considered in these works. The authors in^19^ addressed the impact of a wind generator with multi-types of FDs on the system operation during the normal and contingency states. The maximum SW benefit is the objective function. However, the FDs costs are not considered in the problem formulation.

The applications of SVC and TCSC were presented in^20^ to mitigate the WPC and enhance the WP penetration. The results show the improvement that occurred in the penetration of WP due to the reactive compensation provided by the SVC device. To facilitate WP integration, a comprehensive methodology for the optimal placement of TCSCs and SVCs in a transmission network is proposed in^21^. The cost of WP integration, the cost of generated reactive and active power, and the cost of allocated FDs were considered for a range of operating situations with several probabilistically modeled load growth profiles as well as for the FDs’ lifetimes. Case studies in^22^ have shown that the spillage level can be reduced by making WP a priority in the OPF up to 13.11% for a typical summer and 10.8% for a typical winter day. Also, the results in^22^ show that up to 23% more wind sources can be integrated into electrical power networks due to the implementation of SVC and TCSC. However, the authors in^20,22^ did not consider any from the LS, SW, and FDs costs in their problem formulation.

The authors in^2^, by using an OPF with a fixed series capacitor (FSC) and TCSC, provided a methodology to minimize WPS. In addition, LS is avoided and the total active power losses are minimized as well. The results proved that the effect of the TCSC on mitigating the WPS is more effective than FSC. Two distinct types of FDs consisting of static synchronous compensator (STATCOM) and TCSC are utilized in^23^ to maximize SW and alleviate congestion in the power network at the minimum possible cost. The results in^23^ confirmed that the congestion of the system was not only alleviated by the proposed interactive FDs model but also increased the system flexibility to harvest the WP as much as possible. A novel approach was proposed in^24^ to maximize the utilization of WP by using a combination of STATCOM and shunt capacitor (SC). The results show that the total system flexibility can be affected by the proposed model compared to the system without STATCOM and SC in enhancing the utilization of the WP. The authors in^25^ to minimize WPS, proposed the model of multi-time scale coordinated scheduling with distributed power flow controller. This methodology could be applied to other sorts of FDs to promote the utilization of WP in the power systems. Also, the effects of different types of FDs, such as PST, SVC, and UPFC, are checked and evaluated in^26^ to enhance the total transfer capability, which enables the balance of line flow and regulates node voltage simultaneously. But in these woks, both the LS and the voltage stability are not considered. The cases studied in^27^, and^28^ for optimization of a UPFC and STATCOM have indicated that FDs controllers were able to reduce the costs of congestion. To improve the system loadability and minimize the spillage, several optimal placement methods, and sizing of TCSC, SVC, and UPFC were developed^29^. However, the SW and the costs of FDs are not considered in the problem formulation for the works in^27–29^.

In the presence of substantial renewable energy resources, different FDs impacts on the grid functionality were analyzed. For example, the effect of large-scale WP integration on the security of the power system indices in the presence of FDs was analyzed^30^. In^31^, the authors presented a high capital and maintenance cost. It is mentioned that while the WPC cost might be less than the costs of the FDs, this cost would be greatly exacerbated in a high-penetration wind scenario. Therefore, in the planning studies, the costs and benefits of the FDs should be properly modeled to optimize the power system operation and mitigate wind energy curtailment^14,15^. Additionally, because FDs are expensive, it is essential to put them in the optimal location to enhance network security and improve the voltage stability margin (VSM)^3^.

There are several soft computing methods discussed in the literature that can be used to minimize the WPS such as the krill herd algorithm in^12^, Monte Carlo simulation in^23^, linear programing in^26^, genetic algorithm (GA) in^29^ and so on. However, the WPS issue is nonlinear and non-convex, the optimization algorithm that is used to solve this problem should have a low oscillation in a steady-state, short convergence time, and. low failure rate. Therefore, evaluations of the optimization algorithms should be based on these three factors. For this reason, in this paper, the newly proposed optimization algorithm known as the musical chairs algorithm (MCA) is introduced to enhance the exploration performance by overcoming the deficiencies of the previous methods and improving the three shortcomings mentioned before. The new proposed MCA only requires one parameter, which makes it more simpler to tune than previous algorithms like the krill herd algorithm, which requires three parameters.

Table 1 presents a comparison of models in references adopted in the related literature and proposed methodology in this work.

**Table 1:** Comparison of the models adopted in previous literature and proposed technique.

Ref	Year	Model	Optimization algorithm	Types of FDs			Objectives					Case study
				TCSC	SVC	UPFC	WPS	LS	Cost	SW	VSM	
^2^	2014	Bilevel	OPF model	✓	×	×	✓	✓	×	×	×	24-bus
^11^	2007	Single level		✓	×	×	×	×	✓	✓	×	30-bus
^12^	2016	Single level	Oppositional Krill Herd	✓	×	×	×	×	✓	×	×	
^13^	2018	Bilevel	DC-OPF model	✓	×	×	✓	✓	✓	✓	×	118-bus
^14,15^	2018	Single level	Non-dominated sorting GA	×	✓	×	✓	×	✓	×	×	24-bus
^16^	2010	Single level	Conventional PI and fuzzy controller	×	✓	×	✓	×	×	×	×	3-bus
^17^	2011	Single level	Security Constrained UC	×	×	×	✓	×	×	×	✓	8-bus
^18^	2018	Bilevel	Comprehensive UC model	×	×	✓	✓	✓	✓	×	✓	6-bus
^20^	2016	Single level	Multi-period OPF	✓	✓	×	✓	×	×	×	×	30-bus
^22^	2017	Bilevel	Sequential Monte Carlo simulation	✓	✓	×	✓	×	✓	×	×	96-bus
^23^	2020	Bilevel	Monte Carlo simulation	✓	×	×	✓	×	✓	✓	×	24-bus
^24^	2018	Bilevel	OPF model	×	×	×	✓	×	×	×	×	
^26^	2003	Single level	Linear programming	×	✓	✓	×	×	×	×	×	118-bus
^27^	2007	Bilevel	Interior point OPF	×	×	✓	×	×	×	×	×	30-bus
^29^	2013	Single level	Graphical user interface based on GA	✓	✓	✓	×	✓	×	×	✓	300-bus
Current work		Bilevel	Multi-scenario musical chairs	✓	✓	✓	✓	✓	✓	✓	✓	24-bus

**Figure 1:**
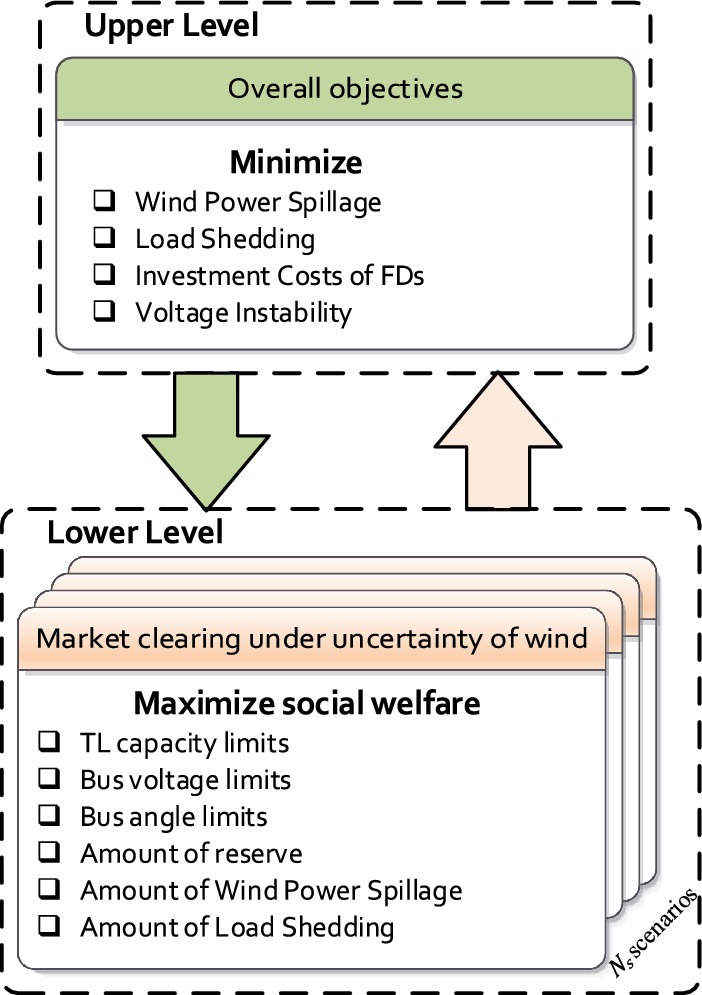
Decision framework of the proposed bilevel model.

This paper proposes a multi-objective bilevel optimization technique based on multi-scenario MCA to minimize WPS, LS, and FACTS costs as well as maximize the SW and the VSM. The proposed bilevel model co-optimize the optimal location and setting of three different types of FDs; TCSC, SVC, and UPFC in the lower-level optimization problem depending on the market clearing under the different scenarios of load demand and WP generation. To regulate the power flow, the power injection/absorbing, phase angle difference, and reactance across the TLs can be varied efficiently by using these FDs. They are suitable for WP integration and reduce the WPS in the power system. The main contribution of this article is to minimize the WPS by obtaining the optimal location and size of FDs based on the conditions of the market clearing under different scenarios of the load and the WP production considering the voltage stability of the system. Based on the optimal size and location of FDs, the amount of WPS and LS will be obtained. The WPS, and LS will be minimized and the VSM will be improved in the upper-level. In addition, the SW will be maximized in the lower-level problem as shown in Fig. 1. A collection of lower-level problem that represent the market clearing conditions can be used to constrain the upper-level problem under different WP and load demand scenarios.

Considering the literature review above, the presented work attempts to provide the following contributions:

1. Proposing a multi-objective bilevel optimization technique based on multi-scenario MCA to minimize WPS, LS, and FACTS costs as well as maximize the SW and VSM.
2. Investigating the optimal allocation and setting of multi-type FDs under multi-scenarios of WP and load demand based on the probabilistic methods.
3. Managing the uncertainty of the WP and load demand by using the Weibull distribution and Gaussian probabilistic methods.

The outline of the article is as follows: The model of WP and load demand and the technique to generate and reduce the scenarios of the load-wind are discussed in detail in Section “Probabilistic modelling of wind power and load demand”. In Section “Modeling of FACTS devices (FDs)”, the injection models of different three types of FDs and the reformulation technique are demonstrated. The description of the voltage stability index is given in Section “Voltage stability index”. Section “Problem formulation” provides the problem formulation of the proposed bilevel model. Based on the decomposition algorithm, the solution approach is demonstrated in Section “Algorithm for the proposed approach”. Section “Case study and results analysis” represents the IEEE RTS 24-bus system as the case study in detail and the numerical results based on this case study. Finally, the conclusion is proposed in Section “Conclusion”.

## Probabilistic modelling of wind power and load demand

This section discusses the uncertainty modeling of the load demand and the WP generation by using the probabilistic method. After the probabilistic method is applied to generate the scenarios for the load demand and the WP generation, a scenario reduction or clustering technique to reduce these scenarios is discussed.

### Wind power generation modelling

**Figure 2:**
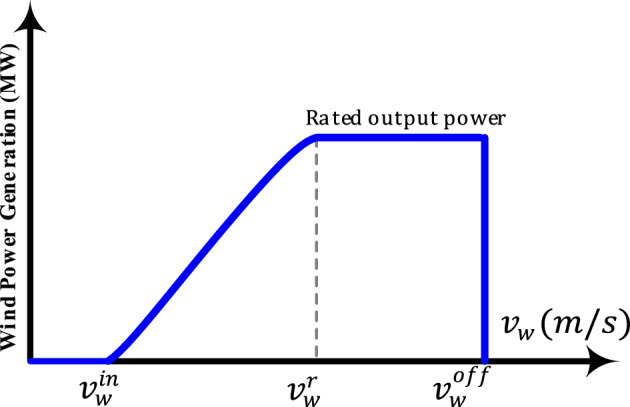
Relationship between the output power and the speed of wind.

By utilizing and installing many wind turbines, wind farms can be established to produce large amounts of power. These wind farms can be connected to the networks of transmission and distribution. In wind farms, different kinds of wind turbines with different parameters and rated capacities are used. The wind farm modeling consists of two main parts: the first one is the wind speed and the second one is the curve of the wind turbine that is used to determine the WP generation depending on wind speed. According to the speed of the wind, the output of the wind farm is nonlinear. The output of the wind farm varies from zero to its rated power due to the change in the wind speed and, hence leads to fluctuations in power flow. Due to that, all the characteristics of the power system such as nodal prices, SW, and powers of TLs are changing. As a result, these uncertainties need to be taken into account while formulating power flow. An excellent approach for addressing this issue is a probabilistic one. Weibull distributions^32^ are a popular model for wind speed, the mean and standard deviation of the wind speed can be used to determine the shape and scale parameters of the distributions. The nonlinear relationship between the wind speeds and the WP outputs is shown in Fig. 2^32^.

Through this, a chronological expression of the wind speeds and WP outputs is made possible. So, the output power of a wind turbine ${P}_{{g}_{w}}({v}_{w})$ for the given wind speed ${v}_{w}$ can be formulated as follows^32^:

1 $${P}_{{g}_{w}}\left({v}_{w}\right)=\left\{\begin{array}{ll}0 & {v}_{w}^{off}\le {v}_{w}\le {v}_{w}^{in}\\ 0.5\rho .A.{C}_{P}\left(\beta ,\lambda \right).{v}_{w}^{3} & {v}_{w}^{in}<{v}_{w}\le {v}_{w}^{r} \\ {P}_{{g}_{w}}^{r} & {v}_{w}^{r}<{v}_{w}< {v}_{w}^{off}\end{array}\right.$$

As a result, a probabilistic model is typically used to describe the power production scenarios of wind turbines. The WPG characteristics can be illustrated by the Weibull distribution. The probability distribution function (PDF) of Weibull can be described as^32^:

2 $$f\left({v}_{w}, {\lambda }_{w},{k}_{w}\right)=\frac{{k}_{w}}{{\lambda }_{w}}{\left(\frac{{v}_{w}}{{\lambda }_{w}}\right)}^{{k}_{w}-1}exp\left(-{\left(\frac{{v}_{w}}{{\lambda }_{w}}\right)}^{{k}_{w}}\right)$$

where ${k}_{w}$ and ${\lambda }_{w}$ stand for shape and scale parameters of the probability density function of the Weibull respectively.

### Probabilistic load demand modelling

**Figure 3:**
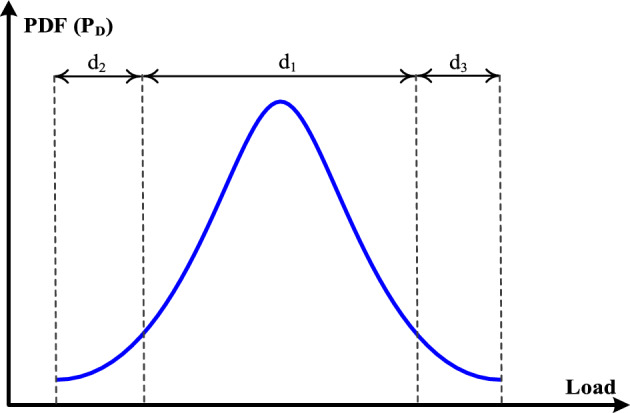
Load PDF and load scenario generation.

It is necessary to describe the uncertainty of the load in the planning and operation of power systems due to the stochastic nature of the load demand. Typically, the normal of the Gaussian PDF can be used to model the uncertainty of the load. The standard and the mean deviation of the load PDF in this paper are assumed to be known. The load scenario probability can be calculated using (3) as shown the Fig. 3^33^.

3 $$f\left({P}_{D}\right)={\int }_{{P}_{D}^{min}}^{{P}_{D}^{max}}\frac{1}{\sqrt{2\pi {\sigma }^{2}}}{e}^{\frac{-({P}_{D}-{\mu }_{D})}{2{\sigma }^{2}}}d{P}_{D}$$

where, $f\left({P}_{D}\right)$ is the load scenario probability, ${P}_{D}^{min}$ and ${P}_{D}^{max}$ are the load scenario boundaries, and $\sigma$ and ${\mu }_{D}$ are the load scenario variance and mean, respectively.

### Clustering or reduction technique

To group the data into sets based on similarities is the main goal of the generic clustering algorithm. The data is based on physical process observations (e.g., the load demand and WP production in the existing electric system). Several algorithms have been proposed in the literature for clustering. Due to the good performance and the simplicity of the K-means algorithm^34^, the authors relied on it in the presented work. The cluster is defined as a group of similar observations that are different from other observations in other clusters. The predicted data in different locations of the studied electric system from the load demand and WP represented the observations. The minimization of the historical observations from the load demand and the WP to a small set of clusters is the main objective of the clustering technique. In addition, by using the mean value of WP production and demand in each location, the centroid of each cluster can be defined. According to these definitions, the next iterative algorithm is described as the K-means algorithm^35^:

- **Step 1:** According to the problem, choose the required number for clusters. Each one is distinguished by the quantity of the initial observations allocated to it as well as the magnitudes of the electrical load and the WP output at various locations.
- **Step 2:** Define each cluster's initial centroid.
- **Step 3:** Calculate the distances between each cluster centroid and each original observation.
- **Step 4:** According to these distances, allocate each historical observation to its cluster.
- **Step 5:** By using the historical observations for each cluster, the cluster centroids can be recalculated.

Steps 3 to 5 are repeated until there are no more changes between two consecutive iterations in the cluster compositions. The cluster centroids and the total number of observations assigned to each cluster are the output of this algorithm. Keep in mind that in each cluster, the number of observations is used to determine the weight of each scenario^36^.

In the literature, several approaches have been proposed to determine the number of clusters for k-mean clustering algorithm such as the rule of thumb; Elbow method; information criterion approach, etc. In the proposed work the authors used the Elbow Method. The main idea of this method is to compute the k-means clustering algorithm for different values of k. For instance, by varying k from 1 to 10 clusters, for each k, calculate the total within-cluster sum of square. Plot the curve of the within-cluster sum of squares according to the number of clusters k. The location of a knee in the plot is generally considered as an indicator of the appropriate number of clusters^35^. In the results, the value of k is equal to 4.

In k-mean, the first centroid is selected randomly from the data points. Once the first centroid is selected, the algorithm looks for the record the furthest in the entire data set. This point becomes the second centroid. Then, for each record, the algorithm computes the distance between the record and the second centroids and keeps the shortest distance of the two. Let's call this value d. The record with the highest d value becomes the third centroid. And so on, until all centroids have been initialized.

## Modeling of FACTS devices (FDs)

As a result of the rapid development of power electronic devices, FDs have become less expensive and more popular. FDs can be modeled in two ways: (i) the model of the power injection/absorb, (ii) the model of impedance insertion. In this study, the FD can be considered by the static power injection model as an element that can inject/absorb active and/or reactive power at terminal buses or TLs in which the device is located^23,36^. As mentioned before, TCSC, SVC, and UPFC are the FDs selected in this paper to help the power system for the minimization of WPS and LS as well as maximization of the SW and improving the VSM of the system. They are chosen due to their low investment costs, ability to increase loadability, and fast control responses. The detailed models for these FDs are presented in the following subsection.

### Modelling of TCSC

**Figure 4:**
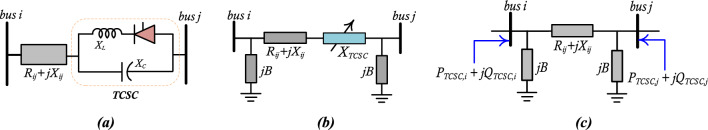
(**a**) basic structure of TCSC, (**b**) Model of TL including TCSC, and (**c**) Power injection model.

TCSC consists of a fixed capacitor and a thyristor-controlled reactor operated in parallel leading to a variable reactance as shown in Fig. 4a. TCSC can be worked as a controllable reactance located in series with the related TL. For the power flow studies, by adding inductive or capacitive impedance, the TCSC varies the overall effective series impedance of the TLs^37,38^. According to this approach, the equivalent circuit of TCSC with the TL can be shown in Fig. 4b. A percentage of the line's reactance value (${X}_{l}$) should be used as the series compensation, to guarantee the system stability. This percentage can be capacitive (${K}_{cap}$) or inductive (${K}_{ind}$). As a result, the new reactance of the TL is calculated as follows after installing the TCSC^38^:

4 $${X}_{ij}={X}_{l}+{X}_{TCSC}$$

5 $${K}_{ind} {X}_{l}\le {X}_{TCSC} \le {K}_{cap} {X}_{l}$$

${X}_{TCSC}$ is set between 0.2 ${X}_{l}$ (inductive) and − 0.7 ${X}_{l}$(capacitive) to avoid the overcompensation^38^. Based on Fig. 4c, the injected active and reactive power due to TCSC are represented in this study by ${P}_{TCSC}$ and ${Q}_{TCSC}$, respectively, and can be calculated at buses $i, j$ as follows^36^:

6 $${P}_{TCSC,i}={V}_{i}^{2}\Delta {G}_{ij}- {V}_{i}{V}_{j} \left(\Delta {G}_{ij}\mathit{cos}\left({\theta }_{ij}\right)+ \Delta {B}_{ij}\mathit{sin}\left({\theta }_{ij}\right)\right)$$

7 $${P}_{TCSC,j}={-V}_{j}^{2}\Delta {G}_{ij}- {V}_{i}{V}_{j} \left(\Delta {G}_{ij}\mathit{cos}\left({\theta }_{ij}\right)- \Delta {B}_{ij}\mathit{sin}\left({\theta }_{ij}\right)\right)$$

8 $${Q}_{TCSC,i}={-V}_{i}^{2}\Delta {B}_{ij}- {V}_{i}{V}_{j} \left(\Delta {G}_{ij}\mathit{sin}\left({\theta }_{ij}\right)- \Delta {B}_{ij}c\mathit{os}\left({\theta }_{ij}\right)\right)$$

9 $${Q}_{TCSC,j}={-V}_{j}^{2}\Delta {B}_{ij}+ {V}_{i}{V}_{j} \left(\Delta {G}_{ij}\mathit{sin}\left({\theta }_{ij}\right)+ \Delta {B}_{ij}\mathit{cos}\left({\theta }_{ij}\right)\right)$$

where

10 $$\Delta {G}_{ij}=\frac{{X}_{TCSC}{R}_{ij} \left({X}_{TCSC}-2{X}_{ij}\right)}{\left({R}_{ij}^{2}+ {X}_{ij}^{2}\right)\left({R}_{ij}^{2}+ {\left({X}_{ij}- {X}_{TCSC}\right)}^{2}\right)}$$

11 $$\Delta {B}_{ij}=\frac{{X}_{TCSC} \left({R}_{ij}^{2}- {X}_{ij}^{2}+{X}_{TCSC}-{X}_{ij}\right)}{\left({R}_{ij}^{2}+ {X}_{ij}^{2}\right)\left({R}_{ij}^{2}+ {\left({X}_{ij}- {X}_{TCSC}\right)}^{2}\right)}$$

### Modelling of SVC

**Figure 5:**
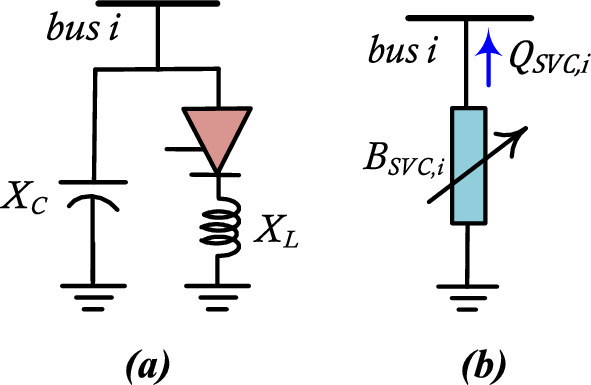
The representation of SVC (**a**) Equivalent circuit, and (**b**) model of the Power flow.

To provide voltage support, SVC is modeled to inject or absorb reactive power as a generator. The SVC when absorbs or injects reactive power, assumes the role of the inductive and capacitive compensators, respectively. It is represented electrically as a shunt element connected to bus $i$ as shown in Fig. 5.

The reactive power provided by SVC can be calculated as given^37^:

12 $${Q}_{SVC,i}={-V}^{2}\times {B}_{SVC,i}$$

where

13 $${B}_{SVC,i}^{ min}\le {B}_{SVC,i }\le {B}_{SVC,i}^{ max}$$

### Modelling of UPFC

**Figure 6:**
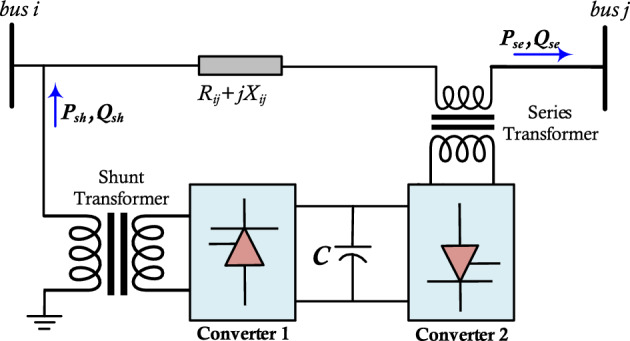
Basic structure of UPFC.

Figure 6 represents the basic model of UPFC. It consists of shunt and series voltage source inverters which share a common DC link. Through two coupling transformers, these two inverters are connected to the power system. The UPFC can be used to control the phase angle, impedance, voltage, and real and reactive power flow in TLs. The shunt converter of UPFC can regulate the voltage drop on the TL whereas the series converter is used to control the power flow^39^. The UPFC can be a decoupled or a coupled model. The UPFC model consists of the two series combinations of an impedance and a voltage source. One of them is connected in series to the TL, while the second is connected in shunt. Through the UPFC control system, the two combinations are coupled. The coupled model is achieved if the change in a set is associated with the change in the other set. While the above two voltage source–impedance combinations are independent in the decoupled model^40^. In the coupled model, due to the modification of the Jacobian matrix, the second model is simpler than the first^39^. So, without the modification of the Jacobian matrix, in the algorithms of the conventional power flow, the implementation of the decoupled model becomes easier.

The power flow of UPFC series branches is shown in (14), and (15) while the power flow of UPFC shunt branches is shown in (16), and (17).

14 $${P}_{se}={V}_{i}^{2}{G}_{ii}- {V}_{i}{V}_{j} \left({G}_{ij}\mathit{cos}\left({\theta }_{ij}\right)+ {B}_{ij}\mathit{sin}\left({\theta }_{ij}\right)\right)-{V}_{i}{V}_{se}\left({G}_{ij}cos\left({\theta }_{i}-{\theta }_{se}\right)+{B}_{ij} sin\left({\theta }_{i}-{\theta }_{se}\right)\right)$$

15 $${Q}_{se}={-V}_{i}^{2}{B}_{ii}- {V}_{i}{V}_{j} \left({G}_{ij}\mathit{sin}\left({\theta }_{ij}\right)- {B}_{ij}\mathit{cos}\left({\theta }_{ij}\right)\right)-{V}_{i}{V}_{se}\left({G}_{ij}sin\left({\theta }_{i}-{\theta }_{se}\right)-{B}_{ij} cos\left({\theta }_{i}-{\theta }_{se}\right)\right)$$

16 $${P}_{sh}={V}_{i}^{2}{G}_{sh}- {V}_{i}{V}_{sh}\left({G}_{sh}\mathit{cos}\left({\theta }_{i}-{\theta }_{sh}\right)+{B}_{sh}\mathit{sin}\left({\theta }_{i}-{\theta }_{sh}\right)\right)$$

17 $${Q}_{sh}={-V}_{i}^{2}{B}_{sh}- {V}_{i}{V}_{sh}\left({G}_{sh}\mathit{sin}\left({\theta }_{i}-{\theta }_{sh}\right)-{B}_{sh}\mathit{cos}\left({\theta }_{i}-{\theta }_{sh}\right)\right)$$

## Voltage stability index

The increasing integration of renewable energy sources into the power system, rapid load changes, and increasing power demand creates problems for the stability of power systems. Many countries have already reported cases of voltage collapse with losses of millions of dollars. This phenomenon is characterized by a progressive decline in voltage magnitudes and occurs basically due to the system's inability to meet the growing demand for reactive power at certain buses in stressed situations. For all these reasons the voltage stability problem has received a lot of attention not only from researchers but also from the industry^41^.

Moreover, it is important to note that none of the studies discussed above take voltage stability issues into account. Power systems are typically run at their stability limitations after reducing the WPS due to the highest utilization of transmission facilities to increase profit in the electricity market. Such a power system can be extremely susceptible to voltage disturbances. Thus, it is essential to reduce the WPS while maintaining sufficient levels of voltage stability. After processing the WPS, an acceptable level of voltage stability must be maintained to make the power system robust against disturbances.

Voltage stability is "the ability of a power system to maintain acceptable voltages at all buses under normal conditions and after being subjected to a disturbance"^42^. Therefore, the voltage stability index becomes an important indicator of power system stability. Voltage stability assessment is a major issue in monitoring the power system stability. Many indices have been proposed in the literature to assess the voltage stability^42,43^. These indices are used for triggering the countermeasures against voltage instability. Some indices are functions of the power system impedance but some others are independent of it and only need the voltage and current of buses. In practice, determining power system impedance is not possible with high precision due to the atmospheric effects and insufficient information about the power system. So, the performance of indices which are functions of power system impedance is always associated with error^44^.

In order to measure voltage stability, the concept of VSM has been proposed to demonstrate the closeness of the current operating point to the point of voltage collapse^44^. The VSM is used in this study as an indicator to check the system's voltage stability. The authors in^42^, and^43^ compared the results of the VSM with those of the L-index, fast voltage stability index, line stability index, new line stability index, and new voltage stability index and found that VSM is the most suitable one in inducting the voltage stability, as most of them depend on system impedance.

**Figure 7:**
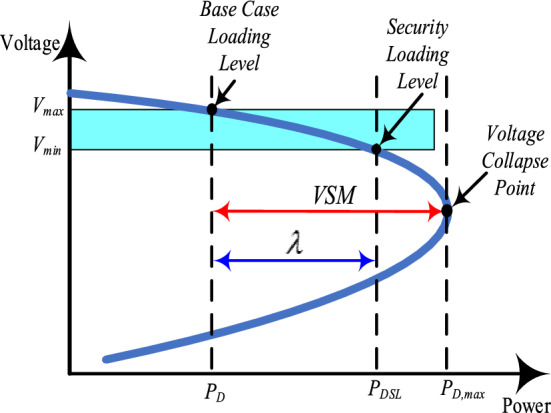
The reaction between loading and bus voltage.

A review of the literature reveals that there are different kinds of techniques for VSM evaluation, such as P–V curve method, V–Q curve method and reactive power reserve, methods based on the singularity of power flow Jacobian matrix at the point of voltage collapse, and the continuation power flow method, etc. In this paper, the VSM is computed as the maximum load increase which can be supplied by the system from the base case loading until it reaches the voltage stability limit. The VSM is obtained by using P–V curve methods^42^. These curves are obtained by considering load increases for all the load busbars of the system as a proportion of the base case loading. The generation level is also increased (in proportion to the base-case injection) to match the load increases during the construction of the P–V curve. For each load increase, a new load-flow problem must be solved, with the set of equilibrium points obtained defining the P–V curve. The stability margin represents the distance, in MW or percentage, from the base case operation point to the point of maximum power transfer capability of the system (P–V curve nose point). The P–V curve and voltage stability margin are illustrated in Fig. 7. The VSM, which is measured in p.u. is given as in (18)^42^:

18 $$VSM=\left({P}_{D.max}- {P}_{D}\right)/{P}_{D}$$

A greater VSM implies a more secure power system and therefore guarantees that not any relatively small disturbance leads to instability of the system and the system is operating far from the voltage instability margin.

## Problem formulation

The proposed multi-objective optimization model to enhance the power system stability, minimize the WPS, LS, and maximize the SW by determining the optimal location and setting of the FDs is presented. It is important to note that the suggested method for enhancement of VSM ensures that the result produced after minimizing the WPS is a secure operating point. As a result, the suggested multi-objective optimization strengthens the power system's resistance to disturbances and lowers the rate of blackouts. A bilevel multi-objective optimization model is formulated to find the optimal location and sizes for multi-type FDs. The main objective functions of the upper-level are to minimize the WPS, LS, and voltage instability. While the lower level is the operation sub-problem, the objective is to maximize the SW by selecting the optimal location and setting of the FDs. The problem of the upper-level is constrained by using a group of the problem of the lower-level, which represents the conditions of the market clearing under the different scenarios of the load demand and WP production.

### Upper-level sub-problem

In the formulation of the upper-level, there are four-objective functions will be proposed. These objective functions are the minimization of the WPS cost, the investment cost of each type of FDs, the total LS cost, and the improvement of the VSM. The first one is calculated as follows^2^:

19 $$\mathit{Min} {f}_{1}= \sum_{s\in {\Omega }^{s}}{\rho }_{s} \sum_{w\in {\Omega }^{W}} {{ C}_{w}^{SP} P}_{ws}^{SP}$$

While the second objective, the annual investment cost of FDs, that can be calculated from the total cost taking into account the lifetime of the device and the interest rate as given in^13^:

20 $$Min {f}_{2}=TC\times \frac{y{\left(1+y\right)}^{LT}}{{\left(1+y\right)}^{LT}-1}$$

where $LT$ is the device lifetime and $y$ is the yearly interest rate. In this paper, $LT$ is selected to be 10 years and $y$ is 10%. While the total FDs cost ($TC$) is given as:

21 $$\mathit{TC}=\sum_{m={\Omega }^{TC}}{S}_{TCSC,m }\times {C}_{TCSC,m }+\sum_{n={\Omega }^{SV}}{S}_{SVC,n }\times {C}_{SVC,n }+\sum_{u={\Omega }^{UP}}{S}_{UPFC,u }\times {C}_{UPFC,u }$$

where

22 $${C}_{TCSC }=0.0015{S}_{TCSC}^{2}-0.713{S}_{TCSC}+153.75$$

23 $${C}_{SVC}=0.0003{S}_{SVC}^{2}-0.3051{S}_{SVC}+127.38$$

24 $${C}_{UPFC}=0.0003{S}_{UPFC}^{2}-0.2691{S}_{UPFC}+188.22$$

The third objective is the minimization of LS cost which is described as follows^2^:

25 $$\mathit{Min} {f}_{3}=\sum_{s\in {\Omega }^{s}}{\rho }_{s}\sum_{d\in {\Omega }^{D}} {C}_{d}^{SH} {P}_{ds}^{SH}$$

Finally, the fourth objective is the maximization of VSM which can be given as^45^:

26 $$\mathit{Max} {f}_{4}=VSM$$

In this work, the multi-objective optimization problem is converted into a single-objective optimization problem by using a penalty factor $h$^46^. This penalty factor converts the VSM quantities into VSM costs. So, the overall objective function of the upper-level can be expressed as in (27).

29 $${F}_{U\_L}={f}_{1}+{f}_{2}+{f}_{3}+h{f}_{4}$$

The maximum penalty rate factor $h$ is defined as the ratio between the highest predictable value of operating cost (${cost}^{max}$) and the highest estimated value for the VSM (${F}_{VSM}^{max}$), which is given by^46^:

28 $$h={cost}^{max}/{F}_{VSM}^{max}$$

### Lower-level sub-problem

The competition between demand and supply is the basic idea of deregulation so that all participants, i.e. distribution companies, generator companies, and the customers maximize their individual welfare. For supplying a specific amount of power, the generator company provided the supply bid which represents the minimum asking price that it would accept. Similarly, for consuming a specific amount of power, the maximum price that the consumer would pay is the demand bid. The task of selling and buying power between generators and distribution companies for customers falls within the responsibility of the system operator based on demand and supply bids to maximize SW. So, the objective function of the lower-level optimization sub-problem is to maximize the SW, which is the difference between the consumer's benefit and the overall cost of the supplier’s power production. The objective function can be computed as follows^47^:

29 $$Max. {f}_{5}=\sum_{s\in {\Omega }^{s}}{\rho }_{s} \left[\sum_{d\in {\Omega }^{D}}{B}_{d}\left({P}_{ds}\right)-\sum_{g\in {\Omega }^{G}}{C}_{p}\left({P}_{gs}\right)\right]$$

where ${C}_{p}\left({P}_{gs}\right)$ is the generator active power production cost, ${B}_{d}\left({P}_{ds}\right)$ is the benefit of the consumer, ${P}_{gs}$ is the generator active power output, ${P}_{ds}$ is the demand active power.

Each thermal generator's and WP real power generation cost function is represented by the following quadratic function^47^:

30 $${C}_{p}\left({P}_{gs}\right)=\sum_{s\in {\Omega }^{s}}{\rho }_{s} \left[\sum_{g\in {\Omega }^{G}}{a}_{g }{P}_{gs}^{2}+ {b}_{g}{P}_{gs}+{c}_{g} +\sum_{w\in {\Omega }^{W}}{d}_{w}{f}_{PDF}(P){P}_{ws}\right]$$

where ${d}_{w}$ is the cost coefficient of the wind turbine, ${f}_{PDF}$ is the probability density function of the wind turbine^48^.

While the benefit of the consumer can be calculated from the following equation^47^:

31 $${B}_{d}\left({P}_{ds}\right)=\sum_{d\in {\Omega }^{D}}{b}_{d}\left({P}_{ds}\right)$$

where ${b}_{d}$ is the slope of the consumer benefit curve.

### System constraints

In the OPF problem, there are two types of constraints to consider. The following equations describe the system constraints.

#### Equality constraints

The equality constraints to be considered are as follows:

32 $$\sum_{g\in {\Omega }^{G}}{P}_{gs}^{r}+\sum_{w\in {\Omega }^{W}}\left({P}_{ws}-{P}_{w}^{fix}-{P}_{ws}^{SP}\right)+\sum_{d\in {\Omega }^{D}}{P}_{ds}^{SH}=\sum_{(i,j)\in {\Omega }^{i}}{P}_{ijs}\left(v, \theta ,{x}^{FDs}\right)-\sum_{\left(i,j\right)\in {\Omega }^{i}}{P}_{ij}^{0}\left(v, \theta \right) , {\forall }_{i,j}, {\forall }_{s}$$

33 $$\sum_{g\in {\Omega }^{G}}{Q}_{gs}^{r}=\sum_{(i,j)\in {\Omega }^{i}}{Q}_{ijs}\left(v, \theta ,{x}^{FDs}\right)-\sum_{\left(i,j\right)\in {\Omega }^{i}}{Q}_{ij}^{0}\left(v, \theta \right) , {\forall }_{i,j}, {\forall }_{s}$$

34 $${\theta }_{\left(i=1\right)s}=0 , {\forall }_{s}$$

Constraints (32) and (33) represent the real and the reactive power balances for the lower level for each scenario and at each bus. The unexpected deviations of wind production are met with the amount of WPS, and/or LS due to these balance constraints^2^. Constraint (34) sets $i$ = 1 as the reference bus.

#### Inequality constraints

The inequality constraints to be considered are as follows:

35 $${v}_{i}^{l}\le {v}_{is}\le {v}_{i}^{u}, \quad \quad {\forall }_{i}, {\forall }_{s} )$$

36 $$-\pi \le {\theta }_{is}\le \pi , \quad \quad {\forall }_{i}, {\forall }_{s}$$

37 $$\left|{S}_{ijs}\left(v, \theta ,{x}^{FDs}\right)\right|\le {S}_{ij,max} , \quad \quad {\forall }_{i}, {\forall }_{j}\in {\Omega }^{i}, {\forall }_{s}$$

38 $${x}_{ij min}^{FDs}\le {x}_{ijs}^{FDs}\le {x}_{ij max}^{FDs} , \quad \quad {\forall }_{i}, {\forall }_{j}\in {\Omega }^{i}, {\forall }_{s}$$

39 $$0\le {P}_{ds}^{SH}\le {P}_{d}^{C} , \quad \quad {\forall }_{d}\in {\Omega }^{D}, {\forall }_{s}$$

40 $$0\le {P}_{ws}^{SP}\le {P}_{ws} , \quad \quad {\forall }_{w}\in {\Omega }^{W}, {\forall }_{s}$$

41 $${-P}_{rg}^{d}\le {P}_{gs}^{r}\le {P}_{rg}^{u} , \quad \quad {\forall }_{g}\in {\Omega }^{G}, {\forall }_{s}$$

42 $${-Q}_{rg}^{d}\le {Q}_{gs}^{r}\le {Q}_{rg}^{u} , \quad \quad {\forall }_{g}\in {\Omega }^{G}, {\forall }_{s}$$

43 $${Q}_{g}^{c}\le {Q}_{gs}^{r}+{Q}_{g}^{0}\le {Q}_{g}^{p} , \quad \quad {\forall }_{g}\in {\Omega }^{G}, {\forall }_{s}$$

Constraints (35) represent the upper and the lower values of the voltage magnitudes, while (36) enforce the angles of buses. The limits on TL capacities are enforced through (37). The reactance of each FD must fall within its upper and lower boundaries according to constraints (38). The value of LS is described by (39) while the value of the WPS is represented by (40). The deployed active and the reactive reserves were constrained by (41) and (42) respectively. The feasibility of each generating unit's production of reactive power is ensured by (43).

## Algorithm for the proposed approach

As can be seen in Fig. 1, the multi-objective function of the upper-level problem depends on the OPF that occur in the lower-level problem. The bilevel programming is represented by the interaction between the decision variables of lower and upper-level problems^49^. This problem was tackled by using many metaheuristic optimization algorithms which used to balance the performances of exploration and exploitation. Through all iterations, a constant number of searching agents is used by these algorithms. To enhance explorations in the first steps of the optimization, the bilevel problem requires high numbers of searching agents, whereas to enhance exploitations in the last stage of optimization, lower numbers of searching agents are required. At the end of the search steps, the searching agents’ number should be minimized to have a lower number of search agents to enhance exploitation. The musical chairs game, which starts with a large number of chairs and players before reducing them one at a time in each round to improve exploration at the beginning of the search and exploitation at the end of the search stages, served as inspiration for this problem^50^. So, in this paper, a novel optimization algorithm known as the multi-scenario MCA is used. Compared to other optimization techniques, the MCA significantly reduced convergence durations and failure rates when solving the bilevel problem.

In the initialization of the MCA, the players are randomly assigned their position or their position is based on any uniform distribution criterion. After that, the fitness value can be obtained from the test system. In each iteration, the loser, which is the player with the lowest fitness value, quits, and the rest of the players are assigned chairs. The chair with the lowest fitness value is also removed at the end of each iteration. The optimization iteration starts after that by adding the searching step to each player, therefore, a new position of the player is given as shown in (44)^51^.

44 $${d}_{P\vartheta }^{g}={d}_{P\vartheta }^{g-1}+M.\frac{|u|}{{\xi }^{1/\beta }}.\left({d}_{best}^{g}-{d}_{P\vartheta }^{g-1}\right)$$

where $g$ is the generation number $g=\left(1, 2, \dots ., ite\right)$, $\vartheta$ is the order of searching agent $\vartheta =\left(1, 2, \dots ., n+1\right)$, $n$ is the number of chairs in each iteration, $M$ is the step size of the MCA which can be determined based on the problem, though it is recommended in much research that $M=$ 1, $\beta$ is the L’evy flight step value which is taken as 1.5^47^, $u$ and $\xi$ are matrices with uniform distributions and their values can be determined as shown in (45).

45 $$u\approx N\left(0,{\sigma }_{U}^{2}\right) and \xi \approx N\left(0,{\sigma }_{\xi }^{2}\right)$$

where the variance of $u$ and $\xi$ can be obtained from (46)

46 $${\sigma }_{u}=\left(\frac{\Gamma \left(1+\beta \right).\mathrm{sin}\left(\pi .\beta /2\right)}{\Gamma \left(\frac{1+\beta }{2}\right).{\beta .2}^{\left(\frac{\beta -1}{2}\right)}}\right) and {\sigma }_{\xi }=1$$

The values of fitness functions for the new positions of the players are determined from the objective function of the test system. The new value of the fitness function is compared to the previous one and the higher value replaces the lower one for each player. The fitness value of each chair is compared to the nearest and the one ahead of players and the highest value is allocated to this chair if it has a higher value than the chair fitness value. When the number of chairs is greater than one, the loser player and the chair with the lowest fitness value will be removed from the set of players and chairs in each iteration. When the number of chairs is one, the process continues without excluding any player or chair. When the iterations finish, the position and fitness value of the last chair are recorded as the global best value. The use of this newly proposed MCA optimization algorithm provides better results than all the other swarm optimization algorithms in terms of convergence time and failure rate, and these results were better than expected^51^.

The MCA requires initialization for the search agents used in the optimization, exactly as all other optimization algorithms. The following steps provide a detailed illustration of the initialization steps:

- **Step 1:** Set the player's initial number.
- **Step 2:** The bounds of each variable will be used to generate random positions for each player.
- **Step 3:** In the objective function (WPS, FDs cost, LS, VSM, and SW), the players positions will be substituted to get for all players the initial fitness.
- **Step 4:** The worst player should be determined
- **Step 5:** The position and value of the worst player will be removed from the set of the players.
- **Step 6:** The positions and values of the chairs can be determined by the rest of the initial positions and values of the players.
- **Step 7:** The worst chair will be determined and removed from the set of chairs.
- **Step 8:** Each player's fitness value is calculated, the new value is compared to the old one to see if it is superior, and the higher value is preserved.
- **Step 9:** The closest two players to each chair are evaluated to see if their fitness levels are higher than this chair's; if not, the chairs' prior values are retained. The closest player with the highest fitness value then takes the chair position and fitness value's place.
- **Step 10:** If the number of the chairs is larger than 1, both the worst chair and player are removed, otherwise no player or chair is removed.
- **Step 11:** The stopping criterion or the end of iterations is checked, otherwise go to step 8.

**Figure 8:**
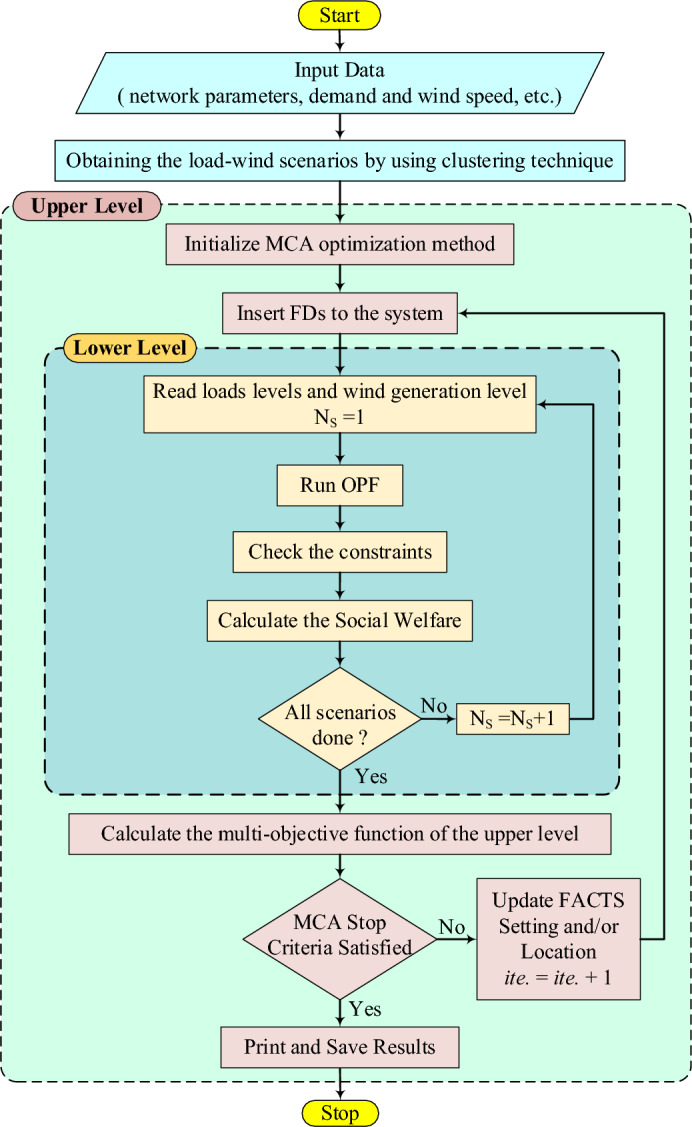
Flow chart of the proposed algorithm.

Based on the optimal size and location of FDs, the amount of WPS and LS will be obtained. The WPS, and LS will be minimized and the VSM will be improved in the upper-level. In addition, the SW will be maximized in the lower-level problem as shown in detail in Fig. 8. The detailed steps of the suggested algorithm are given as follows:

- **Step 1:** Read the system data, load demand wind scenarios, etc.
- **Step 2:** By using the clustering technique, the load-wind scenarios will be reduced.
- **Step 3:** Initialize MCA optimization method.
- **Step 4:** Insert FDs into the system.
- **Step 5:** Read loads and WP generations level for the first scenario (${N}_{s}=1$), the beginning of the lower level.
- **Step 6:** Run OPF and check the constraints.
- **Step 7:** Compute the objective of the lower level (SW), and determine the system variable like WP, LS, VSM, etc.
- **Step 8:** If all the scenarios are done go to step 9, else ${N}_{s}={N}_{s}+1$ and then go to step 5.
- **Step 9:** Calculate the fitness value of each player for all objectives of the upper level; WPS cost, LS penalty, FD costs, and VSM.
- **Step 10:** If the stopping criteria are satisfied, the player associated with the best size and location is the optimal solution. Print and save the results. Otherwise, set *ite* = *ite* + 1 and return to Step 3.

The proposed algorithm will be tested on the modified IEEE 24-bus reliability test system. This system will be described in detail in the next section.

## Case study and results analysis

### Data and system description

**Figure 9:**
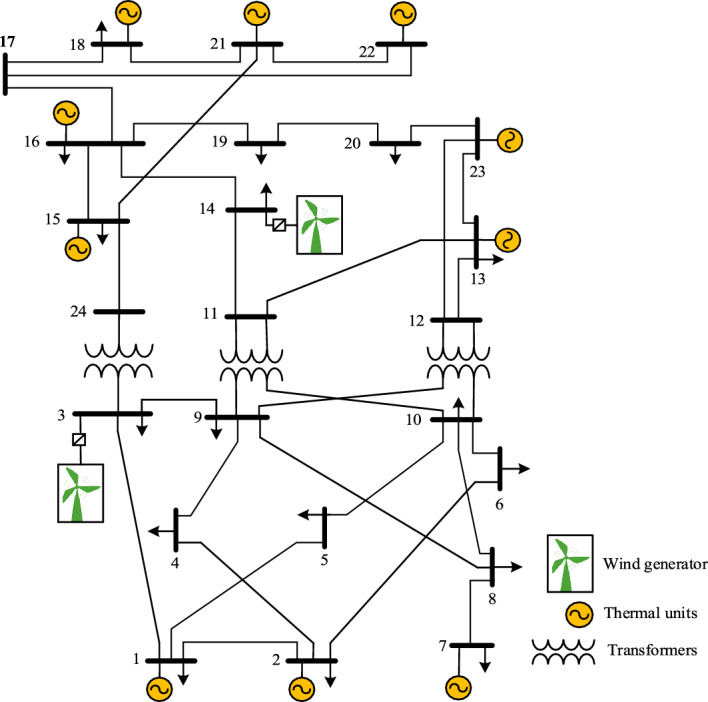
IEEE one area 24 bus RTS.

The proposed methodology in this study is implemented on a modified IEEE-RTS 24-bus test system. This system consists of 32 generators and 34 transmission lines as shown in Fig. 9. The maximum generation and the total load of the system are 3405 MW and 2850 MW, respectively. The bus 13 is selected to be the slack bus. Some modifications have been performed to integrate wind turbines into the system. These modifications are considered as the following:

- To represent the occurrence of the WPS, ratings of the line (From-To) 12–23 were reduced to 220 MW, and the lines 3–24, 15–21, 15–24, and 16–17 were reduced to 175 MW. The ampacity of other TLs is reduced to 90% from their original values.
- The total load of the system is increased by 1.2 from the original one to 3420 MW.
- Two wind farms with a rated capacity of 500 MW for each are installed at Bus 3 and Bus 14 respectively^2^. Each wind farm is different from the other in the amount of power that is produced in each scenario. The wind turbines are assumed to be operated at unity power factor.
- The following data is the characteristics of each wind turbine: the cut-in speed = 3 m/s, the rated speed = 13 m/s, and the cut-out speed = 20 m/s.
- For all buses, the upper and the lower limits of the magnitude of bus voltage are 1.1 p.u. and 0.9 p.u., respectively.
- The cost coefficient of the WPS is selected to be 120$/MWh and the cost coefficient of the LS is selected to be 5000$/MWh.

The ramming simulation parameters are given as in Table 2.

**Table 2:** The simulation parameters.

Parameter	Value
$${X}_{TCSC}$$	− 0.7 $${X}_{l}<{X}_{TCSC}<0.2 {X}_{l}$$
$${B}_{SVC}$$	$$-100<{B}_{SVC }<100$$
$$LT$$	10 years
$$y$$	10%
$${P}_{{g}_{w}}^{r}$$	500 MW
Cut-in speed	3 m/s
Cut-out speed	20 m/s
Rated speed	13 m/s
$${C}_{w}^{SP}$$	120$/MWh
$${C}_{d}^{SH}$$	5000$/MWh
$${v}_{i}^{l}$$	0.9 p.u
$${v}_{i}^{u}$$	1.1 p.u
Population size	50
Iterations	100

Based on the historical data, the probabilistic method is applied to generate the scenarios for the load demand and the WP generation. After that, by using the scenario reduction technique (K-means), the scenarios of the WP and the load demand are reduced from 8760 to 20 scenarios. In the scenario reduction process, scenarios that have low probabilities or are closely related are deleted. For the final 20 scenarios, the amount of the WP in (MW), the levels of the load, and the number of operating hours are provided in Table 3.

**Table 3:** The scenarios of the load demand and WP production*.*

Scenario	No. of hours	Load level	WP1	WP2	Scenario	No. of hours	Load level	WP1	WP2
1	521	0.72096	371	271	11	423	0.96778	250	262
2	653	0.92794	257	103	12	410	0.83615	487	356
3	561	0.51411	474	332	13	391	0.36164	420	368
4	456	1.18792	348	269	14	361	0.58605	170	264
5	340	0.43970	245	426	15	356	0.74167	293	384
6	190	0.32023	233	446	16	552	0.76116	364	298
7	510	0.94090	238	392	17	720	0.15828	154	246
8	572	1.05723	355	284	18	650	0.25319	256	245
9	452	0.70885	491	254	19	101	0.18398	375	296
10	440	0.36164	343	465	20	101	1.19917	439	365

### Numerical results

Table 4 presents the optimal location, setting, and investment costs for the FDs for the different fourteen cases due to the different numbers of installed FDs from the TCSCs, SVCs, and UPFC. The number of the different cases is shown in column 1. The second column represents the different numbers of the FDs used in each case. While the columns from 3 to 11 represent the optimal setting (MVAr) and location and the investment costs (M $) for each type of the FDs from the TCSC, SVC, and UPFC in each case. The last column represents the total investment cost (M $) for all the FDs in the different cases.

**Table 4:** IEEE-RTS 24 bus system results for FDs for different cases.

Cases	FDs	TCSC			SVC			UPFC			TC
		Location	Setting	Cost	Location	Setting	Cost	Location	Setting	Cost	
Case 1	N_T_ = 0 N_S_ = 0 N_U_ = 0	**–**	**–**	**–**	**–**	**–**	**–**	**–**	**–**	**–**	**–**
Case 2	N_T_ = 1 N_S_ = 0 N_U_ = 0	12–13	− 4.36	10.69	**–**	**–**	**–**	**–**	**–**	**–**	10.69
Case 3	N_T_ = 0 N_S_ = 1 N_U_ = 0	–	–	–	8	84.37	142.49	–	–	–	142.49
Case 4	N_T_ = 0 N_S_ = 0 N_U_ = 1	–	–	–	–	–	–	11–13	− 2.36 35.75	7.21 104.14	111.35
Case 5	N_T_ = 2 N_S_ = 0 N_U_ = 0	13–23 9–11	− 3.83 − 4.45	9.41 10.91	–	–	–	–	–	–	20.32
Case 6	N_T_ = 0 N_S_ = 2 N_U_ = 0	–	–	–	11 12	70.34 80.24	122.95 136.89	–	–	–	259.84
Case 7	N_T_ = 1 N_S_ = 1 N_U_ = 0	13–23	− 3.30	8.13	13	74.48	128.87	–	–	–	137.00
Case 8	N_T_ = 1 N_S_ = 1 N_U_ = 1	10–11	− 3.32	8.18	11	56.31	101.86	11–13	− 0.73 29.13	2.23 85.63	197.90
Case 9	N_T_ = 2 N_S_ = 1 N_U_ = 0	13–23 10–11	− 3.88 − 4.31	9.53 10.57	13	30.56	58.85	–	–	–	119.13
Case 10	N_T_ = 1 N_S_ = 2 N_U_ = 0	3–24	− 1.50	3.73	24 8	12.37 53.81	24.89 97.93	–	–	–	126.55
Case 11	N_T_ = 2 N_S_ = 1 N_U_ = 1	3–24 10–11	− 2.21 − 2.01	5.47 4.98	24	21.82	42.92	13–23	− 0.072 39.23	0.2205 113.72	303.46
Case 12	N_T_ = 1 N_S_ = 2 N_U_ = 1	10–11	− 4.06	9.97	12 8	55.81 50.16	101.08 92.11	10–11	− 1.97 20.24	6.02 60.25	312.99
Case 13	N_T_ = 2 N_S_ = 2 N_U_ = 0	13–23 9–11	− 3.43 − 5.98	8.45 14.55	11 8	39.98 81.31	75.25 138.36	–	–	–	269.43
Case 14	N_T_ = 2 N_S_ = 2 N_U_ = 1	13–23 9–11	− 3.38 − 2.98	8.32 7.35	11 8	69.97 68.59	122.41 120.41	10–11	− 2.28 53.16	6.96 151.20	517.04

According to Table 4, the location of the TCSC in case 14 based on all the FDs is the lines 13–23 and 9–11 which is the same in most cases such as cases 5, 7, 9, and case 13. Also, the location of the SVC in case 14 is at buses 11 and 8 like the location in most cases such as 3, 6, 8, 10, 12, and case 13 by using different numbers of the SVC. Finally, the location of the UPFC in case 14 based on all the FDs is 10–11 like the case 12, and the shunt of the UPFC is set at the bus at the receiving end of the TL.

Table 5 presents the results for the fourteen cases due to the different numbers of installed FDs from the TCSCs, SVCs, and UPFC. The total amount of WPS for each wind farm at bus 3 and bus 14 is shown in columns 2 and 3 for each case. The fourth and fifth columns represent the cost of WPS for each wind farm. The sixth and seventh columns show the annual LS amount and the cost of the LS respectively. The eighth column indicates the SW value. Finally, the last column represents the value of the VSM which discussed the improvement in the voltage stability that occurred in the system in each case.

**Table 5:** IEEE-RTS 24 bus system results for objectives of the upper-level for different cases.

Cases	Amount of WPS (MWh)		Cost of WPS (× 10^3^$)		Amount of LS (MWh)	Cost LS (× 10^3^$)	SW (× 10^6^ $)	VSM
	Bus 3	Bus 14	Bus 3	Bus 14				
Case 1	1463.94	865.87	175.67	103.91	1586	7930	98.05	2.769
Case 2	1306.49	796.10	156.78	95.53	1291	6455	98.324	2.833
Case 3	1294.48	602.24	155.34	72.27	1318	6590	98.249	2.807
Case 4	1378.23	587.02	165.38	70.44	1338	6690	98.194	2.794
Case 5	1222.35	569.25	146.68	68.31	988	4940	103.431	2.891
Case 6	1293.65	583.28	155.24	69.99	1275	6375	104.14	2.797
Case 7	1249.75	573.78	149.97	68.85	1025	5125	105.02	2.892
Case 8	1143.76	432.82	137.25	51.94	1146	5730	110.57	3.033
Case 9	1179.35	369.72	141.52	44.37	985	4925	113.63	2.971
Case 10	1180.06	329.70	141.61	39.56	1285	6425	109.96	2.812
Case 11	1076.86	334.18	129.22	40.10	1177	5885	112.93	2.849
Case 12	1039.86	313.08	124.78	37.57	1133	5665	109.64	3.006
Case 13	975.90	385.09	117.11	46.21	916	4580	119.12	2.993
Case 14	900.87	296.05	108.10	35.53	816	4080	120.41	3.199

As concluded from the following Table, the amount of WPS from the wind farm at bus 3 is 1463.94 MWh, and from the wind farm at bus 14 is 865.877 MWh without any FDs and this value decreases to 1222.35 MWh and 569.25 MWh by using two TCSC only. Then, decreases to 1293.65 MWh and 583.28 MWh by using two SVC only. And by using all the FDs which consist of two TCSC, two SVC, and only one UPFC the values decrease to 900.872 MWh and 296.053 MWh for the wind farm at bus 3 and bus 14 respectively. The detailed results are presented in the following Table for each case from the fourteen cases. In addition, by using the suggested number of FDs the LS eliminated from 1586.2 MWh to 816.7 MWh. As the maximum number of TCSC (N_T_), SVC (N_S_), and UPFC(N_U_) increases, the value of each objective function decreases. The voltage stability margin improved from 2.769 to 3.199 and the social welfare increased from 98.05 × 10^6^ MWh to 120.41 × 10^6^ MWh.

The main goal of this paper is to minimize the WPS and to determine from the economic point, whether the more FDs should be installed, or the WP should be curtailed. In addition, the problem of the VSM is one of the main goals. By using more than five devices of the FDs the effect on the WPS becomes poor by comparing it with the last case. So, the maximum number of FDs that can be used to reduce the WPS is selected to be five devices according to the economic view. The best case from the cases that used the FDs is the last case. So, the results of the last case compared to the first case “without the FDs” are shown and explained in detail.

**Figure 10:**
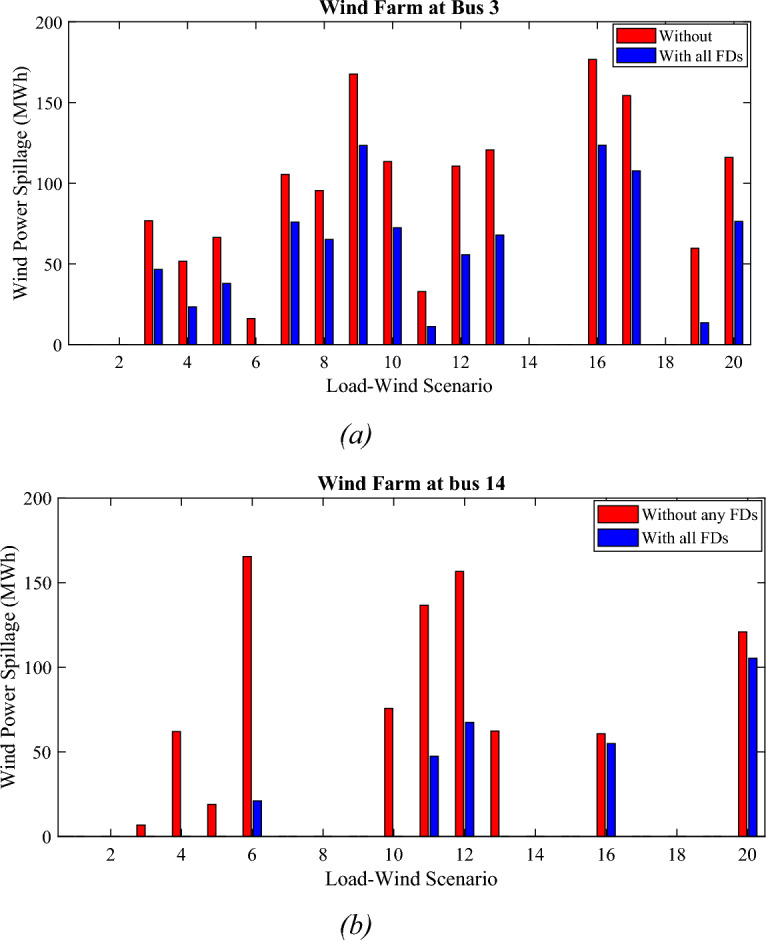
The WPS for each scenario with and without FDs (**a**) WP at bus 3, and (**b**) WP at bus 14.

Figure 10 depicts the WPS for the two wind farms in each scenario in cases one and fourteen. It can be seen that the WPS didn’t occur in all the scenarios for both wind farms at bus 3 and bus 14. The total amount of WPS for the wind farm at bus 3 decreased by 38.46% by using all the suggested numbers of the FDs. Also, the total amount of the WPS for the wind farm at bus 14 decreased by 65.80% due to the use of all the FDs. In scenario number 6 for the wind farm at bus 3 the WPS decreased to zero. Also, scenarios number 3, 4, 5, and 13 for the wind farm at bus 14 decreased to zero due to the use of the FDs.

**Figure 11:**
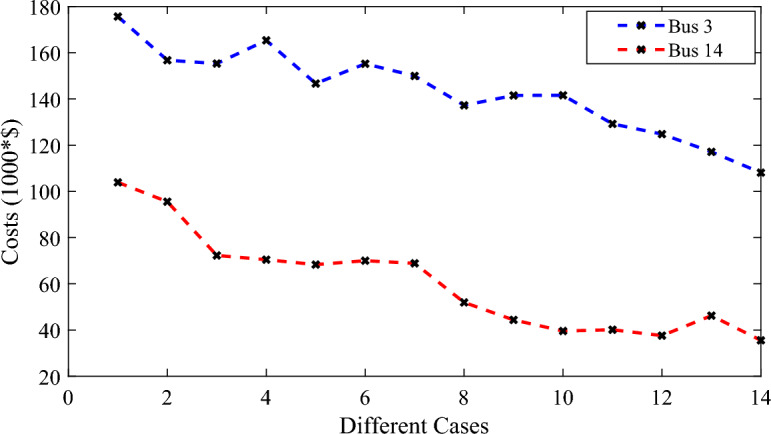
The WPS costs at bus 3 and 14 with the different cases.

Figure 11 represents the costs of the WPS for the wind farm at bus 3 and 14 with different cases by using the different numbers of the FDs. It can be noticed that the number and the type of the FDs effect on the costs of the WPS which reduced in cases more than the other cases. Also, the effect of the number and type of the FDs on the costs of the WPS difference from the wind farm at bus 3 than the wind farm at bus 14.

**Figure 12:**
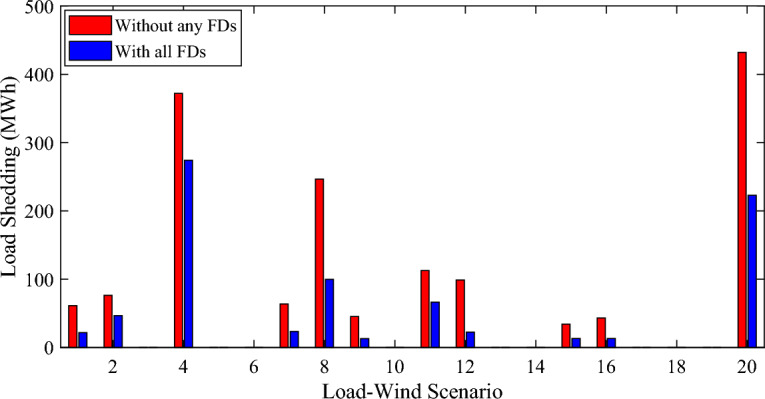
The LS for each scenario with and without FDs.

Figure 12 represents the LS for each scenario. It can be seen that the LS didn’t occur for all the scenarios but there are many scenarios such as 3, 5, and so on where no LS occurs on it. Also, the installation of all the FDs reduces the LS for all the scenarios that the LS occurred on it. The LS decreased by 46.512% by using all the FDs.

**Figure 13:**
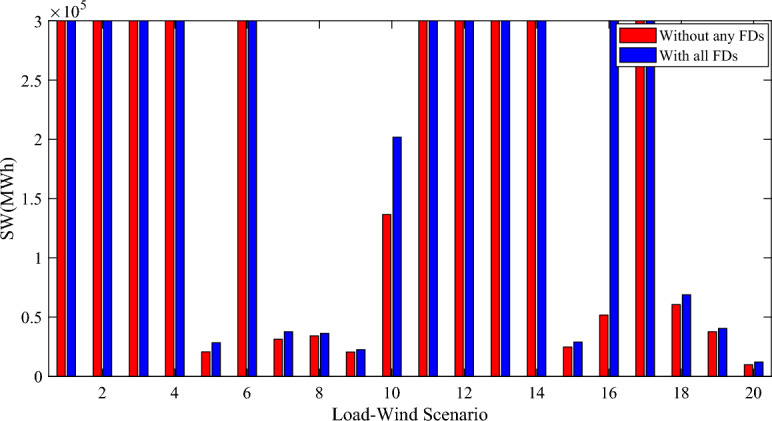
The SW for each scenario with and without FDs.

Figure 13 depicts the SW for each load-wind scenario. It can be seen that by the installation of all the FDs in case fourteen the SW is maximized for all the scenarios. The SW increased by 18.56% due to the use of all the FDs in case fourteen. From the previous Table, it can be noticed that the effect of the different cases by using the different numbers and type of the FDs on the SW.

**Figure 14:**
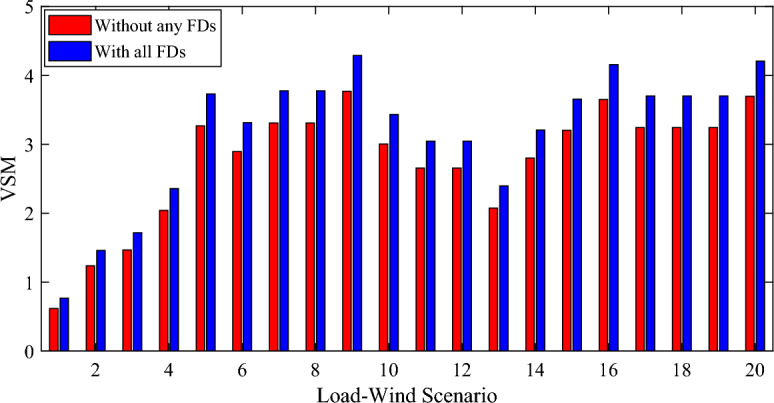
The VSM for each scenario with and without FDs.

Figure 14 represents the VSM for each load-wind scenario in the first case without any FDs and in case fourteen with the installation of all the FDs. It can be seen that in case number fourteen by the installation of all the FDs the VSM is improved for all the scenarios. The VSM improved by 13.44% due to the use of all the FDs in case fourteen. From the previous Table, it can be noticed that the effect of the different cases by using the different number and type of the FDs on the VSM.

**Figure 15:**
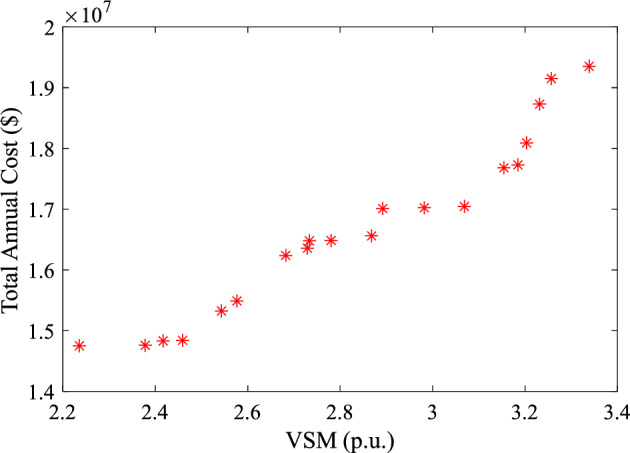
The pareto optimal front curve for the MCA.

The relation between the total annual cost and the VSM (pareto optimal front curve for the MCA) for the case of using all the FDs under the different scenarios of the load demand and WP production is shown in Fig. 15. The figure clearly shows that the relationship is direct between the total annual cost and the increase of system VSM.

Finally, to prove the effectiveness of the proposed algorithm, a comprehensive comparison between the MCA proposed algorithm and the GA is discussed for a selected case in Table 6. The results proved that the proposed algorithm is more effective than the GA where the objectives from the cost of the WPS, and the LS in the case of using the GA is higher than the cost in the case of using the MCA. The PC used in the simulation has an i7-8550U Core, 1.77 GHz CPU, and 8 GB of RAM.

**Table 6:** Comprehensive comparison between MCA and GA.

Cases	Cost of WPS (× 10^3^$)				Cost LS (× 10^3^$)		SW (× 10^6^ $)		VSM		TC of FDs		Time of simulation	
	GA		MCA		GA	MCA	GA	MCA	GA	MCA	GA	MCA	GA	MCA
	Bus 3	Bus 14	Bus 3	Bus 14										
Case 8	158.52	70.89	137.25	51.94	6080	5730	98.24	110.57	2.746	3.033	210.78	197.90	182.36	145.73
Case 12	142.59	54.65	124.78	37.57	5984	5665	99.78	109.64	2.865	3.006	386.78	312.99	278.32	212.86
Case 14	128.78	48.52	108.10	35.53	4250	4080	109.58	120.41	2.982	3.199	589.27	517.04	324.56	267.94

The ramming simulation parameters of the GA are given as in Table 7.

**Table 7:** The simulation parameters of GA.

Parameter	Value
Population size	50
Number of generations	100
Crossover rate	0.90
Mutation rate	0.18

## Conclusion

This paper presented a bilevel model to co-optimize the optimal location and setting of the flexible AC transmission system (FACTS) devices to minimize wind power spillage, so integrate wind energy with maximizing social welfare, and improve the loadability and the voltage stability. In the upper-level problem, the minimization of the wind power spillage, load shedding, and improving the voltage stability margin occurred due to the optimal location and setting of FACTS devices that were achieved in the lower-level. According to this, in the problem of the lower-level, the optimal power flow based on the market clearing is considered under several load-wind scenarios to maximize social welfare. This problem was tackled by a novel optimization algorithm known as the musical chair algorithm. The optimal allocation and setting of multi-type FDs under multi-scenarios of WP and load demand based on the probabilistic methods are discussed. The computational complexity of implementing FDs in the AC-OPF issue is one of the major challenges. The AC-OPF problem using FDs is a large-scale non-convex optimization problem in general. This nonconvexity is due to the nonlinearity of the active and reactive power flow equations. A non-linear solver does not guarantee that a global optimum solution will be obtained, especially when the problem's scale is enormous or during multi-scenario. So, in this paper, the optimal location and setting of FDs are discussed and explained in the presented work.

## Data Availability

The datasets used and/or analysed during the current study available from the corresponding author on reasonable request.

## Abbreviations

FACTS: Flexible AC transmission systems
FDs: FACTS devices
FSC: Fixed series capacitor
FVSI: Fast Voltage Stability Index
GENI: Green electricity network integration
LS: Load shedding
MCA: Musical chairs algorithm
OPF: Optimal power flow
PDF: Probability distribution function
PST: Phase shifter transformer
SC: Shunt capacitor
STATCOM: Static synchronous compensator
SVC: Static VAR compensator
SW: Social welfare
TCSC: Thyristor-controlled series compensator
TLs: Transmission lines
UC: Unit commitment
UPFC: Unified power flow controller
VSM: Voltage stability margin
WP: Wind power
WPC: Wind power curtailment
WPS: Wind power spillage

## Indexes

$d$: Index for demands
$g$: Index for generating units
$i, j$: Indexes for system buses
$l$: Index for TLs
$s$: Index for scenarios
$t$: Index for time periods
$w$: Index for wind farms

## Sets

${\Omega }^{d}$: Set of demands
${\Omega }^{g}$: Set of generating units
${\Omega }^{i}$: Set of buses adjacent to bus $i$
${\Omega }^{s}$: Set of scenarios
${\Omega }^{SV}$: Set of installed SVC
${\Omega }^{TC}$: Set of installed TCSC
${\Omega }^{UP}$: Set of installed UPFC
${\Omega }^{w}$: Set of wind farms

## Parameters and constants

$$A$$: Area covered by the rotor of wind turbine (m^2^)
$${a}_{g },{b}_{g}, {c}_{g}$$: Cost coefficient for generation
$${B}_{ij}$$: Susceptance between buses $$i$$ and $$j$$ (p.u.)
$${B}_{SVC max}$$: Upper bound of SVC susceptance (p.u.)
$${B}_{SVC min}$$: Lower bound of SVC susceptance (p.u.)
$${C}_{d}^{SH}$$: Cost for unserved load ($/MW)
$${C}_{P}$$: Performance coefficient
$${C}_{w}^{SP}$$: Cost coefficient for wind spillage ($/MW)
$${G}_{ij}$$: Conductance between buses $$i$$ and $$j$$ (p.u.)
$${P}_{D.max}$$: Maximum power can transfer to the load (MW)
$${P}_{{g}_{w}}^{r}$$: Wind turbine rated power output (MW)
$${P}_{rg}^{d}$$: Maximum downward reserve active power (MW)
$${P}_{rg}^{u}$$: Maximum upward reserve active power (MW)
$${P}_{w}^{fix}$$: Dispatched power production in market (MW)
$${r}_{ij}$$: Resistance between buses $$i$$ and $$j$$ (p.u.)
$${V}_{i}$$: Voltage magnitude of bus $$i$$ (p.u.)
$${V}_{j}$$: Voltage magnitude of bus $$j$$ (p.u.)
$${v}_{w}^{r}$$: Rated wind speed (m/s)
$${v}_{w}^{in}$$: Cut-in wind speed (m/s)
$${v}_{w}^{off}$$: Cut-off wind speed (m/s)
$${x}_{ij}$$: Reactance between buses $$i$$ and $$j$$ (p.u.)
$${x}_{ij max}^{FDs}$$: Upper bound of the reactance of the FDs (p.u.)
$${x}_{ij min}^{FDs}$$: Lower bound of the reactance of the FDs (p.u.)
$${x}_{l}$$: Transmission line initial reactance (p.u.)
$${x}_{TCSC}$$: Reactance of TCSC (p.u.)
$${\alpha }^{FD}$$: Weighting factor for FDs
$${\alpha }^{SH}$$: Weighting factor for LS
$${\alpha }^{SP}$$: Weighting factor for WPS
$${\alpha }^{VSM}$$: Weighting factor associated with VSM
$${\theta }_{ij}$$: Voltage angle between buses $$i$$ and $$j$$ (p.u.)
$${\theta }_{is}$$: Voltage angle at bus $$i$$ (p.u.)
$$\rho$$: The air density (Kg/m^3^)
$${\rho }_{s}$$: Probability for scenario $$s$$
$$\beta$$: Blade pitch angle (deg)
$$\lambda$$: Tip-speed ratio

## Variables

$${B}_{SVC}$$: Susceptance of installed SVC (p.u.)
$${C}_{SVC}$$: Investment cost of SVC ($/MVar)
$${C}_{Tcsc}$$: Investment cost of TCSC ($/MVar)
$${C}_{UPFC}$$: Investment cost of UPFC ($/MVar)
$${P}_{D}$$: Current load demand operating value (MW)
$${P}_{gs}^{r}$$: Deployed reserve of active power (p.u.)
$${P}_{ds}^{SH}$$: Involuntarily active load shedding (MW)
$${P}_{{g}_{w}}$$: Wind turbine output power (MW)
$${P}_{wavi}$$: Available power of the wind farm (MW)
$${P}_{ws}$$: Output power of wind farm $$w$$ at scenario $$s$$ (MW)
$${P}_{ws}^{SP}$$: Wind power production spillage (MW)
$${Q}_{g}^{0}$$: Initial scheduled reactive power (p.u.)
$${Q}_{gs}^{r}$$: Deployed reserve of reactive power (p.u.)
$${S}_{SVC}$$: Capacities of SVC device (MVar)
$${S}_{Tcsc}$$: Capacities of TCSC device (MVar)
$${S}_{UPFC}$$: Capacities of UPFC device (MVar)
$${v}_{is}$$: Voltage magnitude at bus $$i$$ (p.u.)
$${v}_{w}$$: Wind speed (m/s)
$${x}_{ijs}^{FDs}$$: Reactance of the installed FDs (p.u.)
